# Long Noncoding RNA MIR122HG Inhibits MAVS-Mediated Antiviral Immune Response by Deriving miR-122 in Miiuy Croaker (*Miichthys miiuy*)

**DOI:** 10.3390/v14050930

**Published:** 2022-04-29

**Authors:** Junxia Cui, Weiwei Zheng, Tianjun Xu, Yuena Sun

**Affiliations:** 1 Laboratory of Fish Molecular Immunology, College of Fisheries and Life Science, Shanghai Ocean University, Shanghai 201306, China; cjx371122@163.com (J.C.); zww17805803554@163.com (W.Z.); 2 Laboratory of Marine Biology and Biotechnology, Qingdao National Laboratory for Marine Science and Technology, Qingdao 266000, China; 3 Key Laboratory of Exploration and Utilization of Aquatic Genetic Resources, Shanghai Ocean University, Ministry of Education, Shanghai 201306, China; 4 National Pathogen Collection Center for Aquatic Animals, Shanghai Ocean University, Shanghai 201306, China

**Keywords:** long noncoding RNA, microRNA, MAVS, antiviral immunity, fish

## Abstract

Long noncoding RNAs (lncRNAs) function as micro regulators to impact gene expression after multiple pathogen infections, which have been largely studied in the last few years. Although lncRNA studies on lower vertebrates have received less attention than those on mammals, current studies suggest that lncRNA plays an important role in the immune response of fish to pathogen infections. Here, we studied the effect of MIR122HG as the host gene of miR-122 and indirectly negatively regulate MAVS-mediated antiviral immune responses in miiuy croaker (*Miichthys* *miiuy*). We found that poly(I:C) significantly increases the host MIR122HG expression. The increased MIR122HG expression inhibited the production of the antiviral immune-related genes IFN-1, ISG15 and Viperin upon SCRV treatment. In addition, MIR122HG can act as a pivotally negative regulator involved in the MAVS-mediated NF-κB and IRF3 signaling pathways, which can effectively avoid an excessive immune response. Additionally, we found that MIR122HG can promote the replication of SCRV. Our study provides evidence about the involvement of lncRNAs in the antiviral immune response of fish and broadens the understanding of the function of lncRNAs as a precursor miRNA in teleost fish.

## 1. Introduction

Innate and acquired antiviral immune responses are important for host survival during viral infection. Upon the recognition of viral components, host cells are activated to produce type I interferon (IFN) and pro-inflammatory cytokines, which exert pleiotropic attenuation effects on viral replication in the neighboring cells [1,2]. The production of IFN and pro-inflammatory factors needs to be strictly controlled to attain an appropriate immune response to invading pathogens without causing an immune disorder [3]. The host recognizes the pathogen infection via pattern recognition receptors (PRRs), including Toll-like receptors (TLRs), RIG-I-like receptors (RLRs), Nucleotide oligomerization domain-like receptors (NLRs), and C-type lectin receptors (CLRs). TLRs and RLRs are crucial for the detection of viral RNA [4]. RLRs function differently from the TLRs, which are the cytoplasmic sensors for viral infection, in mediated antiviral responses [5]. Upon effective recognition, RLRs, both RIG-I and MDA5, contain caspase recruitment domains (CARDs) that interact with the crucial adaptor protein mitochondrial antiviral signaling protein (MAVS, also known as IPS-1/CARDIF/VISA), and then the signal is transmitted to the kinases TBK1, inducible IκB kinase (IKKi) and to IκB kinase beta (IKKβ), which activates the interferon regulatory factor 3 (IRF3) and the NF-κB pathway [6,7]. NF-κB and IRF3 were translocated into the nucleus, where they produce inflammatory cytokines and type I interferon, respectively, resulting in a family of IFN-stimulated gene (ISG) production, which generates an eliminatory effect on the viral invasion [8,9]. To ensure anormal immune response, the signaling activities of RLRs are firmly controlled by a multi-step regulatory mechanism [10,11]. Moreover, viruses have formed a myriad of regulation strategies to evade and resist the host immune response for their survival [12]. Therefore, it is important to research the detailed mechanisms of viral clearance and host preservation in the antiviral immune response.

MicroRNAs (miRNAs) are a class of endogenous, highly conserved, noncoding, and small (18–25 nt) RNAs that function mainly as crucial post-transcriptional regulators to regulate gene regulation by binding to the 3′-untranslated region (3′-UTR) of target mRNAs, either suppressing translation or inducing degradation [13,14,15]. In addition, miRNAs play significant roles in the regulation of cellular processes, including cell proliferation and differentiation, apoptosis, viral infections, and carcinogenesis [16,17,18,19]. Since their initial discovery, in mammals hundreds of different miRNAs have been identified, and to some extent up to 60% of all mRNAs are modulated by miRNAs [20]. Previous researchers have demonstrated that some host miRNAs can affect viral replication in the RIG-I signaling pathway at various levels. For example, the expression of miR-146a was induced by vesicular stomatitis virus (VSV) infection, is shown in a RIG-I-dependent signaling pathway, which then by targeting IL-1R-associated kinases 1 and 2 and TNFR-associated factor 6 (TRAF6) inhibits the production of type I IFN sequentially to promote VSV replication [21]. Moreover, miR-29a suppresses the antiviral response by targeting the IFNα receptor to decrease its downstream signaling during VSV infection [22]. Moreover, recent studies have indicated that human miR-122 significantly facilitated hepatitis C virus (HCV) replication, whereas miR-122 by targeting NDRG3 inhibits hepatitis B virus (HBV) replication in human hepatocellular carcinoma (HCC) and HCC-derived cell lines [23,24]. Accumulating evidence has demonstrated the crucial role of miRNAs in regulating RLR-mediated antiviral immunity, whereas the regulation mechanism of adaptor MAVS by miRNA remains poorly understood.

Long noncoding RNA (lncRNA) is made up of a variety of noncoding functional transcripts with a length of more than 200 nucleotides, and has attracted much attention because of its involvement in gene expression. LncRNA can regulate mRNAs through different biological mechanisms. Some lncRNAs can act as endogenous competitive RNAs (ceRNAs) and regulate mRNA expression by competing with mRNA for miRNA, while some lncRNAs can act as host genes carrying microRNAs, which are responsible for producing mature miRNAs, and then suppress the expression of target mRNA [25,26,27,28]. It has been widely report in mammals that lncRNA, as the host gene, participates in the modulation of miRNA targets. For example, MIR100HG indirectly regulates GATA6 by promoting the cotranscription of miRNA-125b to participate in a double-negative feedback loop, providing a new target for cetuximab resistance treatment [29]. LncRNAGm2044 and its product, miR-202, could repress the proliferation of the human testicular embryonic carcinoma cell line NCCIT by targeting Rbfox2 [30]. However, little is known about whether this regulatory mechanism exists in teleost fish.

As a lower vertebrate, fish are an excellent biological model for studying the origin of innate immune response. The innate immune response is the most fundamental defense mechanism for fish to recognize PAMPs and is important as it protects against infection by various pathogens [31]. Rhabdoviruses are a group of single-stranded, enveloped, and negative-sense RNA viruses. In the past few decades, many rhabdoviruses have been determined and isolated from aquaculture fish, including sinipercachuatsi rhabdovirus (SCRV), hematopoietic necrosis virus (IHNV), hirame rhabdovirus (HIRRV), spring viremia of carp virus (SVCV), and viral hemorrhagic septicemia virus (VHSV) [32]. These isolated rhabdoviruses have been reported to cause severe hemorrhagic septicemia in many marine and freshwater fish species [33]. Thus, it is important to study the mechanism of antiviral immune response to virus treatment in teleost fish.

In this study, we further found that the immune-related lncRNA MIR122HG, which acts as the host gene of miR-122, can inhibit MAVS and negatively regulate MAVS-mediated antiviral immune responses in miiuy croaker. We found that poly(I:C) significantly increased the host MIR122HG expression. Moreover, the increased MIR122HG expression inhibited the production of the antiviral immune-related genes IFN-1, ISG15 and Viperin upon SCRV treatment. Furthermore, we demonstrated that MIR122HG could negatively regulate the antiviral immune response by indirectly targeting MAVS and inhibiting the MAVS-mediated NF-κB and IRF3 signaling pathways, which can effectively avoid an excessive immune response. Additionally, we found that MIR122HG can promote the replication of SCRV. Taken together, our study not only provides new insights into understanding the importance of lncRNA in the antiviral immune response, but also reveals an lncRNA–miRNA network in fish.

## 2. Materials and Methods

### 2.1. Animals

Miiuy croakers were obtained from Zhoushan Fisheries Research Institute. Fishes (~50 g) were acclimated at 25 °C for several weeks before experiments [34,35]. Experimental procedures were conducted as described previously [36,37]. All animal experimental procedures were conducted in accordance with the National Institutes of Health’s Guide for the Care and Use of Laboratory Animals, and the experimental protocols were approved by the Research Ethics Committee of Shanghai Ocean University (No. SHOU-DW-2018-047).

### 2.2. Cell Culture and Treatment

EPC cells were cultured, briefly, in medium 199 (Hyclone), containing 10% fetal bovine serum (FBS, Gibco), 100 mg/mL streptomycin, and 100 U/mL penicillin under 5% CO_2_ at 28 °C, as described previously [38]. HEK293 cells were cultured in DMEM high-glucose medium (HyClone) containing 10% FBS, 100 mg/mL streptomycin and 100 U/mL penicillin 5% CO_2_ at 37°C [39]. Additionally, *M. miiuy* kidney cell lines (MKCs) and M. miiuy intestine cell lines (MICs) were cultured in L-15 medium (HyClone) supplemented with 15% FBS, 100 U/mL penicillin, and 100 μg/mL streptomycin at 26 °C, as described previously [40]. For stimulation experiments, MKCs were challenged with poly(I:C) (10 μg/mL) or SCRV with a multiplicity of treatment (MOI) of 5 and harvested at different times for RNA extraction [41].

### 2.3. RNA Oligoribonucleotides

miR-122 mimics (dsRNA oligonucleotides), miR-122 inhibitors (single-stranded oligonucleotides chemically modified by 2′-Ome), the MIR122HG-specific small interfering RNA (si-MIR122HG), and control oligonucleotides were commercially synthesized and acquired from GenePharma (Shanghai, China). Their sequences are as follows: miR-122 mimics, 5′-UGGAGUGUGACAAUGGUGUUUG-3′ (sense) and 5′-AACACCAUUGUCACACUCCAUU-3′ (antisense); miR-122 inhibitors, 5′-CAAACACCAUUGUCACACUCCA-3′; si-MIR122HG, 5′-GGUAUGAAUGAAUGAAUCUTT-3′; negative control (NC or si-NC), 5′-UUCUCCGAACGUGUCACGUTT-3′ (sense) and 5′-ACGUGACACGUUCGGAGAATT-3′ (antisense); inhibitor control, 5′-CAGUACUUUUGUGUAGUACAA-3′.

### 2.4. Plasmids Construction and Transfection

The wild- or mutant-type 3′-UTR region of *M. miiuy*, as well as *D. rerio*, *L. crocea* MAVS gene reporter vector, and the MAVS expression plasmid were constructed as previously described [25]. To construct the MIR122HG expression plasmid, the full-length MIR122HG sequences of the above-mentioned species were cloned into the pcDNA3.1vector, respectively. Additionally, the mutated MIR122HG with deficiency mutations in the mature miR-122region and the mutated reporter plasmids with point mutations in the miR-122 binding site were synthesizedusing the Mut Express II Fast Mutagenesis Kit V2 with specific primers [42]. In addition, M. miiuy MAVS-3′UTR or its mutanttype was cloned into the mVenus-C1 (Invitrogen, Carlsbad, CA, USA), which contains the sequence of an enhanced green fluorescent protein (GFP), as previously described [25]. All the recombinant plasmids were extracted from Endotoxin-Free Plasmid DNA Miniprep Kit (Tiangen, Beijing, China) and confirmed by Sanger sequencing [43,44]. The sequences of all primers are listed in Supplementary Table S1.

Before transient transfection, the cells were seeded into 24-well or 12-well plates and incubated for 24 h. Then, the cells were transfected with the plasmids using Lipofectamine^TM^ 3000 (Invitrogen, Carlsbad, CA, USA), or RNA oligoribonucleotides by using Lipofectamine^TM^ RNAi MAX (Invitrogen, Carlsbad, CA, USA), according to the manufacturer’s instructions. For luciferase experiments, miRNA mimics (100 nM) or miRNA inhibitor (100 nM) and pmirGLO reporter vector (500 ng per well) containing the wild- or mutant-type plasmid of MAVS 3′-UTR were transfected into cells. For functional analyses, the overexpression plasmid (500 ng per well) or control vector (500 ng per well) and miRNA mimics (100 nM), miRNA inhibitor (100 nM), or siRNA (100 nM) were transfected into cells in culture medium and then harvested for further experiments.

### 2.5. RNA Extraction and Real-Time Quantitative PCR

Total RNA and small RNAs (< 200 nt) were isolated with TRIzol Reagent (Invitrogen, Carlsbad, CA, USA) and miRcute miRNA IsolationKit (Tiangen, Beijing, China), respectively, following the manufacturer’s instructions [45]. The cDNA of the total RNA was synthesized using the FastQuant RT Kit (Tiangen, Beijing, China), which includes DNase treatment of RNA to eliminate genomic contamination, and miRcute miRNA FirstStrand cDNA Synthesis Kit (Tiangen, Beijing, China) was applied for the reverse transcription of miRNAs [46,47]. The expression patterns of each gene were performed by using SYBR Premix ExTaq^TM^ (Takara, Japan) [48]. The expression analysis of miR-122 was executed by using the miRcute miRNA qPCR Detection Kit (Tiangen, Beijing, China). Real-time quantitative PCR (qPCR) was performed in an Applied Biosystems QuantStudio 3 (Thermo Fisher Scientific, Waltham, MA, USA), as we described previously [49]. The primers for these genes and miRNAs are shown in Supplementary Table S1. β-actin and 5.8S rRNA were applied as the internal controls in the relative expression level of mRNA/lncRNA and miRNA, respectively.

### 2.6. Dual-Luciferase Reporter Assays

For miRNA target identification, EPC cells and HEK293 cells were cotransfected with wild- or mutant-type MAVS 3′-UTR luciferase reporter, together with MIR122HG or pcDNA3.1. Moreover, to determine the functional regulation of MAVS, EPC cells were cotransfected with NF-κB, Interferon regulatory factor 3 (IRF3), IFN-stimulated response element (ISRE) or type I interferon (IFN-1) luciferase reporter plasmid [25,49], pRL-TK Renilla luciferase plasmid, and MAVS expression plasmid, together with either MIR122HG, MIR122HG-MT or pcDNA3.1, miR-122 mimics or NC, and inhibitors or inhibitor control for dual-luciferase reporter assay. Then, the cells were collected and lysed for reporter activity assays using a dual-luciferase reporter assay system (Promega Madison, WI, USA), according to the manufacturer’s instructions. Among the experiments, the luciferase activity values were measured and compared to the *Renilla* luciferase control. In each experiment, all samples were performed in triplicate, and three independent experiments were performed.

### 2.7. Western Blotting

After 48 h transfection, the cellular lysates were generated by using 1 × SDS-PAGE loading buffer. Using a BCA Protein Assay Kit (Vazyme, Nanjing, China), the soluble protein concentrations was measured and an equal amount of protein was loaded for SDS-PAGE (10%) gel and transferred onto the PVDF (Millipore, Billerica, MA, USA) membranes (Pall Corporation, New York, NY, USA) in semidry blotting (Bio-Rad Trans-Blot Turbo Transfer System). Membranes were blocked with 5% BSA. Membranes were subsequently incubated with anti-Flag mouse (Beyotime, Shangha, China), anti-GAPDH (Beyotime, China), anti-Tubulin (Beyotime, China), or anti-GFP mouse (Beyotime, China) monoclonal antibodies or polyclonal anti-MAVS (Absin, China) antiserum at 4 °C overnight. The antibody against MAVS was diluted to1:500; anti-Flag, anti-Tubulin, and anti-GAPDH monoclonal antibodies were diluted to1:2000; the anti-GFP monoclonal antibody was diluted to1:1000; and HRP-conjugated anti-rabbit IgG or anti-mouse IgG to1:5000 (Abbkine, Redlands, CA, USA). The next day, the membranes were incubated for 60 min with the secondary antibody (Beyotime, China) conjugated with horseradish peroxidase at room temperature. The results are representative of three independent experiments. The immunoreactive proteins were detected by using Western BrightTM ECL (Advansta, Menlo Park, CA, USA) and digital imaging was conducted with a cold CCD camera (Bio-Rad, Hercules, CA, USA).

### 2.8. Statistical Analysis

All experiments were repeated at least three times. Relative gene expression data were obtained using the 2^−∆∆CT^ method and comparisons between groups were analyzed by one-way analysis of variance (ANOVA) followed by Duncan’s multiple comparison tests [50]. The results are expressed as mean ± standard error, and differences between means were considered statistically significant and indicated by * *p* < 0.05 or ** *p* < 0.01 [51,52].

## 3. Results

### 3.1. MIR122HG Inhibits the Host Antiviral Immune Response

**Figure 1:**
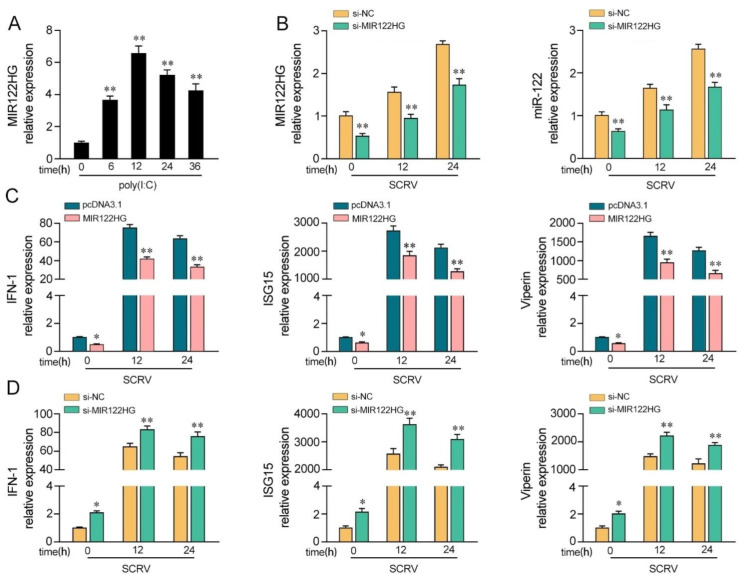
MIR122HG inhibits the host antiviral immune response. (**A**) Expression profiles of MIR122HG in MKCs were measured by qPCR at the indicated time after poly(I:C) stimulation. (**B**) The siRNA of MIR122HGwas transfected into MKCs for 24 h and then treated with SCRV for12 or 24 h. The expression levels of MIR122HG and miR-122 were measured by qPCR. (**C**) MKCs were transfected with MIR122HG or pcDNA3.1 for 24 h and then treated with SCRV for 12 or 24 h.The expression levels of IFN-1, ISG15 and Viperin were measured by qPCR. (**D**) MKCs were transfected with si-MIR122HG or si-NC for 24 h and then treated with SCRV for 12 or 24 h. The expression levels of IFN-1, ISG15 and Viperin were measured by qPCR. All data are presented as the means ± SE from at least three independent triplicate experiments. **, *p* < 0.01; *, *p* < 0.05 for the controls.

Our previous study demonstrated that the expression of MIR122HG is dramatically increased in M. miiuy spleen samples and spleen cell (MSpC) under SCRV treatment [42]. Here, we detected whether the virus-triggered upward trend of MIR122HG existed forMKCs upon poly(I:C) stimulation. As shown in Figure 1A, the results demonstrated that MIR122HG levels were increased significantly upon poly(I:C) stimulation. Furthermore, to investigate the biological functions of MIR122HG, the MIR122HG expression plasmid was constructed and a small interfering RNA (siRNA) of MIR122HG was designed as previously described [42]. Meanwhile, we also measured the effect of the siRNA on the expression of miR-122. As shown in Figure 1B, the results demonstrated that the siRNA of MIR122HG inhibited the expression levels of MIR122HG and miR-122 byvarying degrees upon SCRV treatment. Then, we further explored the role of MIR122HG in antiviral immunity through the overexpression and knockdown of MIR122HG in MKCs under SCRV treatment. Additionally, IFN-1, ISG15 and Viperin are important indicators for detecting the antiviral immune response; thus, the effects of MIR122HG on the IFN-1, ISG15 and Viperin were assayed. The results showed that the overexpression of MIR122HG inhibits the expression of IFN-1, ISG15 and Viperin in MKCs under different SCRV treatment times (Figure 1C). On the contrary, the knockdown of MIR122HGincreases these indicated gene expression levels upon SCRV treatment in MKCs (Figure 1D). Taken together, these data demonstrate that MIR122HG negatively regulates the antiviral immune response.

### 3.2. MIR122HG Inhibits Miiuy Croaker MAVS Gene

**Figure 2:**
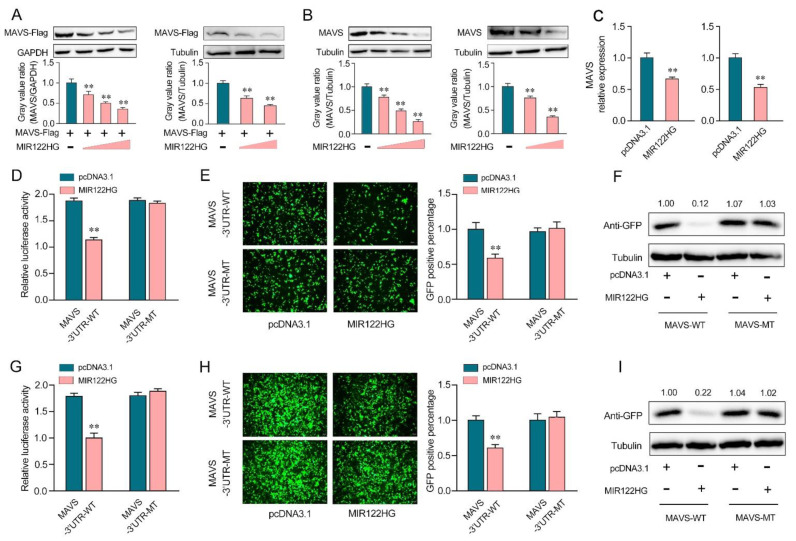
MIR122HG inhibits miiuy croaker MAVS gene. (**A**) EPC cells (left panel) were cotransfected with the MAVS expression plasmid with a flag tag, together with MIR122HG plasmid (0, 100, 200, and 400 ng) or pcDNA3.1 (400, 300, 200, and 0 ng). After 48 h transfection, MAVS expression was measured by Western blotting (upper panel), and the gray value ratio about MAVS/GAPDH (left panel) was shown in the lower panel. HEK293 cells (right panel) were cotransfected with the MAVS expression plasmid with a flag tag, together with MIR122HG plasmid (0, 200, and 400 ng) or pcDNA3.1 (400, 200, and 0 ng). After 48 h transfection, MAVS expression was measured by Western blotting (upper panel), and the gray value ratio about MAVS/Tubulin was shown in the lower panel. (**B**) MKCs (left panel) were transfected with MIR122HG plasmid (0, 100, 200, and 400 ng) or pcDNA3.1 (400, 300, 200, and 0 ng) for 48 h, MAVS expression was determined by Western blotting (upper panel), and the gray value ratio about MAVS/Tubulin was shown in the lower panel. MICs (right panel) were transfected with MIR122HG plasmid (0, 200, and 400 ng) or pcDNA3.1 (400, 200, and 0 ng) for 48 h, MAVS expression was determined by Western blotting (upper panel), and the gray value ratio about MAVS/Tubulin was shown in the lower panel. (**C**) MKCs (left panel) and MICs (right panel) were cotransfected with MIR122HG or pcDNA3.1. After 24 h of transfection, the expression levels of MAVS were determined by qPCR. (**D**) EPC cells were cotransfected with MIR122HG or pcDNA3.1, along with MAVS 3′-UTR-WT or MAVS 3′-UTR-MT for 24 h, and then the luciferase activity was measured. (**E**,**F**) MIR122HG or pcDNA3.1was cotransfected with the wild- or mutant-type mVenus-MAVS 3′-UTR into EPC cells, respectively. At 48 h post-transfection, the fluorescence intensity (**E**) and the GFP expression (**F**) were evaluated by enzyme-labeled instrument and Western blotting, respectively. (**G**) HEK293 cells were cotransfected with MIR122HG or pcDNA3.1, along with MAVS 3′-UTR-WT or MAVS 3′-UTR-MT for 24 h, and then the luciferase activity was measured. (**H**,**I**) MIR122HGor pcDNA3.1wascotransfected with the wild- or mutant-type mVenus-MAVS 3′-UTRinto HEK293 cells, respectively, for 48 h transfection; the fluorescence intensity (**H**) and the GFP expression (**I**) were evaluated by enzyme-labeled instrument and Western blotting. The scale bar represents 20 μm; original magnification, ×10. The luciferase activity was analyzed and normalized to *Renilla* luciferase activity. All data are presented as the means ± SE from at least three independent triplicate experiments. **, *p* < 0.01 forthe controls.

Our previous study demonstrated that miR-122 was able to target the miiuy croaker MAVS gene. To further detect the effect of MIR122HG on the expression of MAVS, we applied a MAVS expression plasmid and transfected it with MIR122HG or pcDNA3.1 into EPC cells (left panel) and HEK293 cells (right panel). As shown in Figure 2A, the results showed that the overexpression of MIR122HG dramatically decreased the MAVS protein level in EPC cells (left panel) and HEK293 cells (right panel). Meanwhile, we detected the role of MIR122HG in endogenous MAVS expression. The results showed that MAVS expression was decreased significantly after the transfection of MIR122HG in MKCs (Figure 2B, left panel) and MICs (Figure 2B, right panel). Meanwhile, we transfected MIR122HG in MKCs and MICs for 48 h, and the expression levels of MIR122HG were detected by the qPCR. The results demonstrated that MIR122HG dramatically inhibited the expression level of MAVS in MKCs (Figure 2C, left panel) and MICs (Figure 2C, right panel). These results preliminarily demonstrated the inhibitory effect of MIR122HG on MAVS expression at the post-transcriptional level.

To further demonstrate that this negative mechanism is achieved through the complementation of MIR122HG with the seed sequences in the MAVS 3′-UTR region, a dual-luciferase report analysis was conducted. As shown in Figure 2D, the luciferase reporter assays showed that the transfection of MIR122HG led to a dramatic decrease in the luciferase activity of the wild-type MAVS 3′-UTR in EPC cells, while there is no change in the mutant-type MAVS 3′-UTR, which contains mutations in the putative binding sites. Additionally, we inserted the wild- or a mutant-type form of MAVS 3′-UTR into the mVenus-C1 vector and examined whether cotransfection with MIR122HG could inhibit the expression levels of green fluorescent protein (GFP) [25]. As shown in Figure 2E,F, the results demonstrated that MIR122HG could dramatically suppress the levels of GFP, demonstrating the interaction between MIR122HG and MAVS. Similar results were observed for the cotransfection with MIR122HG in HEK293 cells (Figure 2G–I). In conclusion, our results strongly demonstrate that MIR122HG can directly inhibit the miiuy croaker MAVS gene.

### 3.3. MIR122HG Negatively Regulates MAVS-Mediated NF-κB and IRF3 Signaling

**Figure 3:**
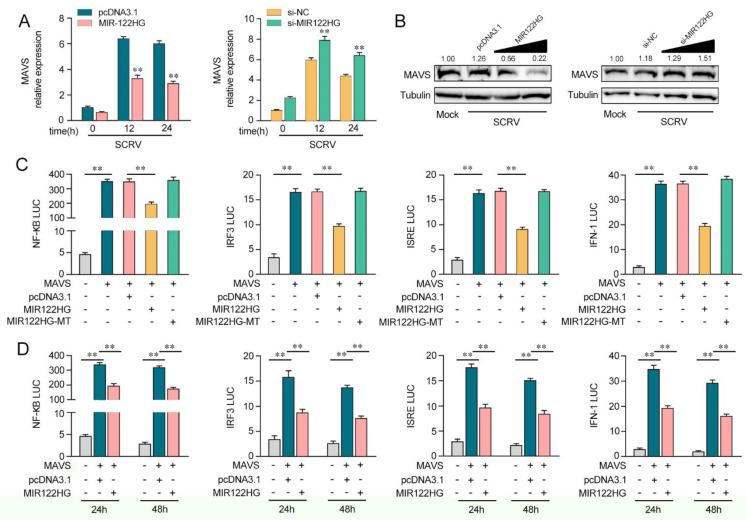
MIR122HG can negatively regulate MAVS-mediated NF-κB and IRF3 signaling. (**A**) The qPCR assays were conducted to measure the expression levels of MAVS in MKCs by transfection with MIR122HG or pcDNA3.1 and si-MIR122HG or si-NC upon SCRV treatment. (**B**) MKCs were cotransfected with MIR122HG (0, 100, 200, and 400 ng) or pcDNA3.1 (400, 300, 200, and 0 ng) and si-MIR122HG (0, 25, 50, and 100 nM) or si-NC (100, 75, 50, and 0 nM) at different concentrations for 24 h, and then the cells were treated with SCRV for another 24 h and the protein levels of MAVS were measured by Western blotting. (**C**) EPC cells were cotransfected with MIR122HG, MIR122HG-MT or pcDNA3.1, along with MAVS expression plasmid, pRL-TK vector, NF-κB, IRF3, ISRE or IFN-1 luciferase reporter genes to investigate the regulatory role of MIR122HG in NF-κB and IRF3 signaling. (**D**) EPC cells were cotransfected with MIR122HG or pcDNA3.1, MAVS expression plasmid, and pRL-TK vector, together with NF-κB, IRF3, ISRE or IFN-1 luciferase reporter genes for 24 and 48 h, and then the luciferase activity was determined. Luciferase activity was normalized to *Renilla* luciferase activity. All data are presented as the means ± SE from at least three independent triplicate experiments. **, *p* < 0.01 for the controls.

As miR-122 can regulate MAVS-mediated antiviral pathways, we probed whether MIR122HG, as the host gene of miR-122, can regulate the MAVS-mediated NF-κB and IRF3 signaling pathways. As shown in Figure 3A, the analysis of qPCR showed that the increased expression of MAVS induced by SCRV was attenuated to a certain extent by MIR122HG in MKCs (left panel). However, the knockdown of MIR122HG caused an increase in the expression level of MAVS upon SCRV treatment (Figure 3A, right panel). Additionally, as shown in Figure 3B, MIR122HG significantly decreased the MAVS protein level upon SCRV treatment in a dose-dependent manner, while the silenced MIR122HG promoted the level of MAVS expression at the protein levels in MKCs. These results demonstrate that MIR122HG could regulate MAVS expression at the post-transcriptional level. To determine whether MIR122HG could inhibit MAVS-mediated NF-κB and IRF3 signaling, we conducted dual-luciferase reporter assays. As shown in Figure 3C, MIR122HG inhibits the luciferase reporter activity induced by MAVS-activated NF-κB, IRF3, ISRE and IFN-1 reporter genes, but not by MIR122HG-MT. Then, the dual-luciferase reporter assays were measured for 24 and 48 h of transfection (Figure 3D). Collectively, these data demonstrated that MIR122HG can inhibit the MAVS-mediated NF-κB and IRF3 signaling pathways.

### 3.4. miR-122 Inhibits the MAVS-Mediated Antiviral Signaling Pathway

**Figure 4:**
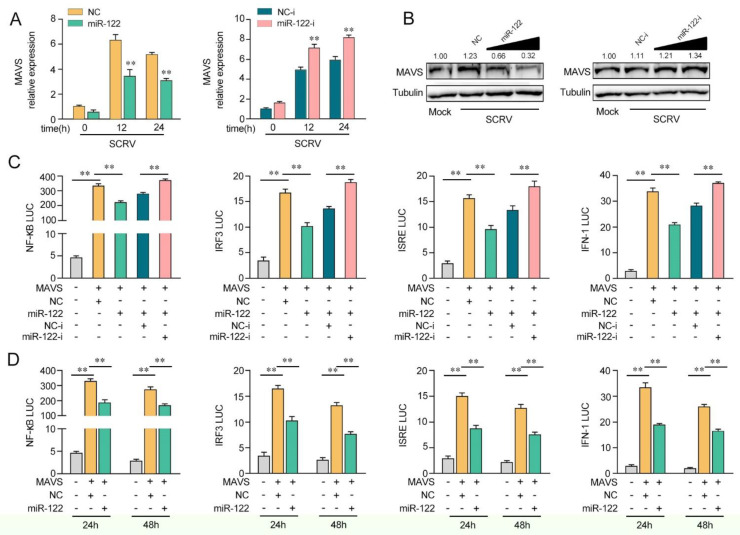
miR-122 is able to suppress MAVS-mediated antiviral signaling. (**A**) The qPCR assays were conducted to measure the expression levels of MAVS in MKCs by transfection with miR-122 or NC, miR-122-i, or NC-i upon SCRV treatment. (**B**) MKCs were cotransfected with miR-122 (0, 25, 50, and 100 nM) or NC (100, 75, 50, and 0 nM) and miR-122-i (0, 25, 50, and 100 nM) or NC-i (100, 75, 50, and 0 nM) for 24 h. Then, the cells were treated with SCRV for another 24 h, and the protein levels of MAVS were measured by Western blotting. (**C**) EPC cells were cotransfected with miR-122 or NC and miR-122-i or NC-i, along with MAVS expression plasmid, pRL-TK vector, NF-κB, IRF3, ISRE, or IFN-1 luciferase reporter genes to investigate the regulatory role of miR-122 on NF-κB and IRF3 signaling. For each transfection, the total amount of oligonucleotides was controlled and normalized (final concentration, 100 nM). (**D**) EPC cells were cotransfected with miR-122 or NC, MAVS expression plasmid, and pRL-TK vector, together with NF-κB, IRF3, ISRE, or IFN-1 luciferase reporter genes for 24 and 48 h, and then the luciferase activity was determined. Luciferase activity was normalized to *Renilla* luciferase activity. All data are presented as the means ± SE from at least three independent triplicate experiments. **, *p* < 0.01 for the controls.

Given that miR-122 targets and regulates MAVS, we probed whether miR-122can regulate MAVS expression upon SCRV treatment. As shown in Figure 4A, upon SCRV treatment, the overexpression of miR-122 inhibited MAVS mRNA expression levels in MKCs, whereas the transfection of miR-122 inhibitors (miR-122-i) increased the mRNA level of MAVS expression. Then, we validated whether miR-122could regulate the expression levels of MAVS expression in MKCs at the protein level upon SCRV treatment. As shown in Figure 4B, the MAVS protein level was dramatically increased after SCRV treatment and the transfection of the miR-122 mimics decreased the MAVS protein level in a dose-dependent manner, whereas the miR-122 inhibitors (miR-122-i) increased the level of MAVS expression. Next, we further probed whether miR-122 regulates MAVS-mediated NF-κB and IRF3 signaling pathways. As shown in Figure 4C, the results showed that MAVS could activate NF-κB, IRF3, ISRE, and IFN-1 luciferase reporter genes. The transfection of miR-122 mimics markedly suppressed the activation of NF-κB, IRF3, ISRE, and IFN-1 luciferase reporter genes induced by the overexpression of MAVS compared with negative control mimics (NC). However, the inhibition effect could be attenuated when the cells were cotransfected with miR-122-i. Then, the dual-luciferase reporter assays were measured at 24 and 48 h post-transfection, and the inhibition effect induced by the transfection of miR-122 mimics was shown to be more dramatic at 24 h post-transfection (Figure 4D). Taken together, these data demonstrate that miR-122 plays as a negative regulator in modulating MAVS-mediated NF-κB and IRF3 signaling pathways.

### 3.5. MIR122HG Enhanced SCRV Replication

**Figure 5:**
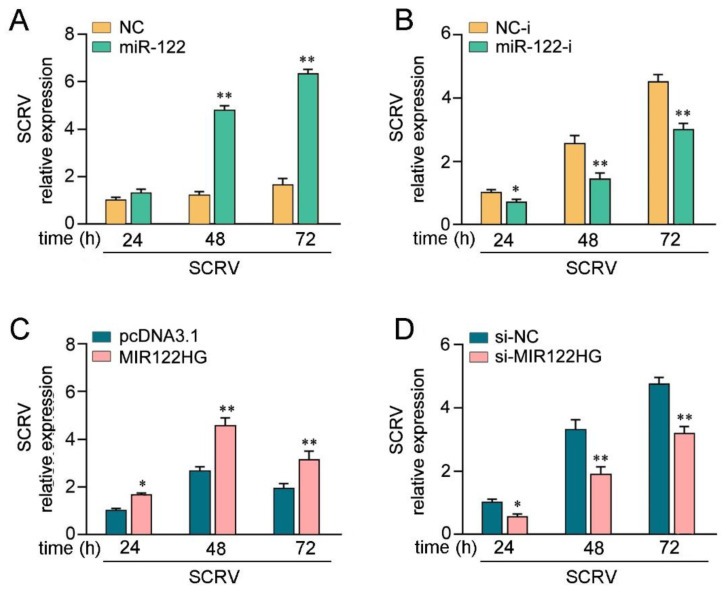
LncRNA MIR122HG promotes SCRV replication. (**A**,**B**) MKCs were transfected with miR-122 or NC (**A**), miR-122-i or NC-i (**B**) for 24 h and then treated withSCRV at different times.The SCRV level in the intracellular was measured by qPCR. (**C**,**D**) MKCs were transfected with MIR122HG or pcDNA3.1 (**C**), si-MIR122HG, or si-NC (**D**) for 24 h and then SCRV treated at adifferent time. The SCRV level in the intracellular was determined by qPCR. All data are presented as the means ± SE from at least three independent triplicate experiments. **, *p* < 0.01; *, *p* < 0.05 for the controls.

To investigate the biological significance of SCRV-triggered upregulated MIR122HG against viral infection in host cells, the effect of MIR122HG on SCRV replication in MKCs was measured. Before examining the effect of MIR122HG on virus proliferation, we first tested whether mature miR-122 could regulate virus replication. By measuring the SCRV levels intracellularly from the infected MKCs, we found that the overexpression of miR-122 promoted SCRV replication, whereas the miR-122 inhibitor decreased SCRV replication (Figure 5A,B). At the same time, the effect of MIR122HG on SCRV replication was measured. This result indicates that MIR122HG can also promote SCRV replication, while the knockdown of MIR122HG caused a decrease in the SCRV replication (Figure 5C,D). Taken together, these results strongly demonstrate that the overexpression of MIR122HG promotes SCRV replication in MKCs.

### 3.6. MIR122HG Regulating MAVS Is Found in Other Teleost Fish

**Figure 6:**
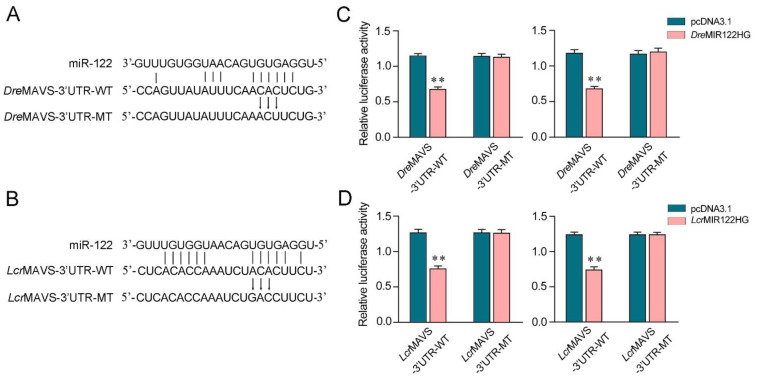
MIR122HG-regulating MAVS gene is widely found in fish. (**A**,**B**) Schematic diagram of the predicted target sites of miR-122 in the 3′-UTR of *D. rerio* MAVS (**A**) and *L. crocea* MAVS (**B**). (**C**) EPC cells (left panel) and HEK293 cells (right panel) were cotransfected with *Dre*MIR122HG or pcDNA3.1, together with the wild-type *Dre*MAVS-3′UTR (WT) or the mutant-type *Dre*MAVS-3′UTR (MT) for 24 h, and then the luciferase activity was measured. (**D**) EPC cells (left panel) and HEK293 cells (right panel) were cotransfected with *Lcr*MIR122HG or pcDNA3.1, together with the wild-type *Lcr*MAVS-3′UTR (WT) or the mutant-type *Lcr*MAVS-3′UTR (MT) for 24 h, and then the luciferase activity was measured. All data are presented as the means ± SE from at least three independent triplicate experiments. **, *p* < 0.01 for the controls.

We aimed to investigate the generality of the finding that MIR122HG could regulate MAVS 3′-UTR in different fish species. First, the miR-122 binding site in MAVS 3′-UTR also displayed high conservation in *D. rerio* and *L. crocea* (Figure 6A,B). To obtain direct evidence that MIR122HG could regulate MAVS 3′-UTR in *D.* *rerio* and *L. crocea*, the luciferase report genes were generated by cloning MAVS 3′-UTR of *D.* *rerio* and *L. crocea* into the pmir-GLO vector, within the devoid of miR-122 binding site as a negative control. Meanwhile, we constructed the MIR122HG expression plasmid of *D.* *rerio* and *L. crocea*. Strikingly, the luciferase activities were decreased when cotransfecting *Dre*MIR122HG or *Lcr*MIR122HG with the wild-type *D.* *rerio* MAVS 3′-UTR or *L. crocea* MAVS 3′-UTR into EPC cells, whereas it showed no effect on the luciferase activity when cotransfected with their mutant types (Figure 6C,D, left).The same experiment was conducted in HEK293 cells again and we obtained similar results (Figure 6C,D, right), which demonstrates that MIR122HG could also target *D.* *rerio* and *L. crocea* MAVS 3′-UTR. Taken together, these results indicate that the precursor of miR-122, MIR122HG, can also exert an inhibitory effect to regulate the expression of the MAVS gene in other species.

## 4. Discussion

**Figure 7:**
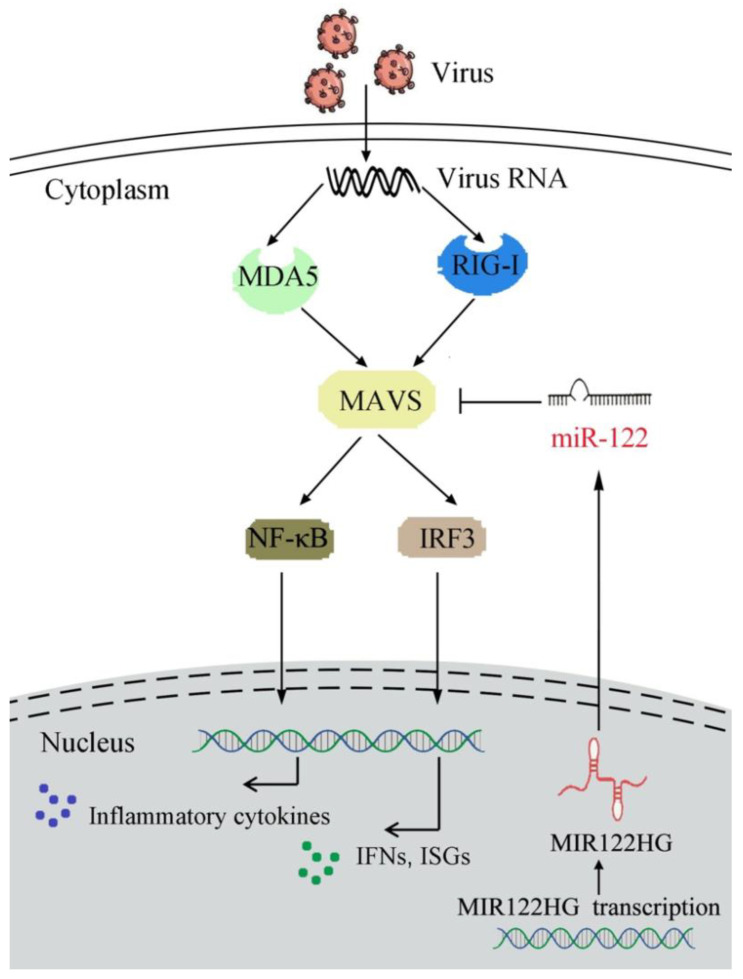
A mechanism model of MAVS regulatory functions and networks. Fish MAVS could induce the innate antiviral immune response by recruiting NF-κB and IRF3 to trigger inflammatory cytokines and the activation of antiviral factors upon SCRV treatment. MIR122HG regulates the release of miR-122 and acts as the precursor of miR-122 to indirectly inhibit MAVS expression and negatively regulates MAVS-mediated NF-κB and IRF3 signaling, which may promote the virus escape.

In recent years, the fish culture has become the most efficient industry in agriculture production. However, the development of fish is hampered by various pathogens, especially viral diseases, which are frequently reported in aquaculture animals [53]. Therefore, it is important to study the underlying regulatory mechanisms of resistance to viral infection in aquaculture species. In this study, we found that MIR122HG negatively regulates the antiviral immune response in teleost fish. We found that MIR122HG functions as the pre-miR-122, the overexpression of which can inhibit the level of MAVS and suppress the MAVS-mediated antiviral innate immune response, thus promoting SCRV replication in fish. Additionally, the present study showed that the MAVS-mediated antiviral signaling pathways were activated and participated in the RNA virus-induced immune regulation network in miiuy croaker. Further research demonstrated that MIR122HG has a regulatory effect on precursor miRNA, which is an important regulator of MAVS and negatively regulates the MAVS-mediated NF-κB and IRF3 signaling pathways (Figure 7). In addition, miR-3570 can inhibit the MAVS-mediated antiviral immune response by targeting MAVS in miiuy croaker upon RNA viral infection [54]. The regulation of MAVS by miR-3570 and miR-122 is both coordinated and independent, as the two miRNAs play a role through different sites on the 3′-UTR of MAVS. However, the regulation of MAVS by the two miRNAs is independent, because miRNAs can regulate mRNA translation by combining their own seed sequence with the 3′-UTR of mRNA, whereas the sequences of miR-3570 and miR-122 are completely different. The two miRNAs we found, miR-3570 and miR-122, both inhibit MAVS expression post-transcriptionally and resist the infection of SCRV, indicating that the two miRNAs can coordinate the regulation of MAVS expression.

miR-122 is a liver-specific miRNA, making up 70% of the miRNAs in the liver. Additionally, miR-122 is highly expressed in mouse and human livers, primary cell-cultured hepatocytes and liver cell lines [55,56]. MiR-122 plays a crucial role in cell differentiation, proliferation, metabolism, and other hepatocyte processes by negative regulating the expression of target genes [13,57,58]. Previous studies have demonstrated thatmiR-122inhibits hepatitis B virus (HBV) replication by targeting NDRG3 in hepatocellular carcinoma cells (HCC) or HCC-derived cell lines [59]. miR-122 can indirectly regulate MDA5 by targeting the *M. miiuy* DAK gene [60]. In addition, studies have demonstrated that IκBα is the target of miR-122, and it can decrease IκBα at the mRNA and protein expression levels [61].

In this work, a novel lncRNA regulatory mechanism was discovered, and it was found that lncRNAs can be negatively regulated and participate in antiviral immune response as miRNA precursors to avoid an excessive immune response and maintain immunity balance. In mammals, a large number of studies have demonstrated that lncRNAs can act as miRNA host genes to regulate miRNA production and indirectly regulate mRNA. For example, in the prognosis of patients with gastriccardia adenocarcinoma (GCA), miR-770 and its host gene MEG3 may play a tumor suppressor role and hypermethylation in the proximal promoter and enhancer regions [62]. Additionally, as the precursor RNA, lncRNAMIR155HG generates two mature miRNAs, miR-155-5p and miR-155-3p, which play a key role in promoting glioma progression and act as a prognostic factor for the survival of patients with glioblastoma [63]. Although it is common for lncRNAs to play a regulatory role as miRNA precursors in mammals, they are still rare in lower vertebrates. In this study, it was identified that MIR122HG can be upregulated upon poly(I:C) stimulation. The overexpression of MIR122HG could inhibit the expression of the antiviral immune-related genes IFN-1, ISG15 and Viperin upon SCRV treatment. In addition, MIR122HGcan act as a pivotally negative regulator involved in the MAVS-mediated NF-κB and IRF3 signaling pathways, which can effectively avoid an excessive immune response. Collectively, these data revealed that miiuy croaker MIR122HG can be a negative regulator, participating in the fish antiviral immune response by indirectly targeting MAVS, which is an effective molecule to control immunity.

ncRNAs are a large number of ncRNAs transcripts encoded by eukaryotes and play an important role in regulating gene expression at the post-transcriptional level. As one of the main members of ncRNA, miRNAs have also received extensive attention due to their regulatory function. Additionally, the function of miRNAs has been gradually studied in various biological processes of many model organisms. In recent years, more and more research on miRNAs in low vertebrates has been carried out, such as those in fish [64]. For instance, our previous research demonstrated that miR-3570 plays a regulatory role in the bacteria-induced inflammatory response by the MyD88-mediated NF-κB signaling pathway via targeting MyD88 [65]. In addition, miR-217 regulates the antibacterial and antiviral immune responses by the TAK1-mediated NF-κB and IRF3 signaling pathways through targeting TAK1 upon the bacteria and SCRV infection [66]. In the present study, we found that MIR122HG could negatively regulate the expression of MAVS and inhibit the MAVS-mediated innate antiviral immune response. Taken together, our finding demonstrated that MIR122HGinhibitsthe expression of MAVS and the MAVS-mediated NF-κB and IRF3 signaling pathways (Figure 7).

In summary, this study showed that MIR122HG, as the reservoir of miR-122, can regulate the adapter molecule MAVS and negatively regulate the MAVS-mediated NF-κB and IRF3 signaling pathways. We found that poly(I:C) significantly increased the expression of MIR122HG. Additionally, the increased expression of MIR122HG inhibited the production of antiviral immune-related genes IFN-1, ISG15 and Viperin upon SCRV treatment. In addition, MIR122HGcan act as a pivotal negative regulator involved in the MAVS-mediated NF-κB and IRF3 signaling pathways, which can effectively avoid an excessive immune response. Additionally, we found that MIR122HG can promote the replication of SCRV. Taken together, these findings demonstrated that lncRNA acts as anegative regulator in the SCRV-triggered immunity response in fish and enriches the network of the host–virus interaction.

## Data Availability

All data analyzed or generated during this study are included in the manuscript.

## Supplementary Materials

The following supporting information can be downloaded at: https://www.mdpi.com/article/10.3390/v14050930/s1. Table S1: PCR primer information in this study.
