# Supplementary material for: Long Noncoding RNA MIR122HG Inhibits MAVS-Mediated Antiviral Immune Response by Deriving miR-122 in Miiuy Croaker (Miichthys miiuy)
Source: Viruses. 2022 Apr 29;14(5):930. doi: 10.3390/v14050930 (PMC9143459; doi:10.3390/v14050930)
Supplement: Supplementary file 1 [file viruses-14-00930-s001.zip › viruses-1688034-supplementary.pdf]

Supplementary Table S1. PCR primer information in this study.

Primers	Sequences (5'-3')
MIR122HG-RT-F	AAACTTGAGGTCGTCCAT
MIR122HG-RT-R	AACTGAGGTCCTTTTCAC
MAVS-qRT-F	AGGCACCAACAATTCCAG
MAVS-qRT-R	ACGGAGCAGGCTTCACTT
IFN-1-qRT-F	TACGATGGCTAATAACTCC
IFN1-qRT-R	CATTGACAAAGTGCTCCA
ISG15-qRT-F	TGAACGGACAGAAGACGC
ISG15-qRT-R	TGAGGAATACCTGCATGG
Viperin-qRT-F	ACCCGTCCAAGTCCATAC
Viperin-qRT-R	TCATGTCAGCTTTGCTCC
SCRV-qRT-F	GGGCTGGATGATAGACGATTG
SCRV-qRT-R	TGGCGGAGGTGCTTGATATGG
miR-122-qRT-F	CGAGTGGAGTGTGACAATGG
miR-122-qRT-R	CAGTTTTTTTTTTTTTTTCAAAC
5.8S rRNA-qRT-F	CTCTTAGCGGTGGATCACT
5.8S rRNA-qRT-R	AGTTTTTTTTTTTTTTTGCCGAGT
β-actin-qRT-F	GAGCCGCACGCTTCTTT
β-actin-qRT-R	CTGCTGTAGCCGAGGAC
MAVS-F	GACGATGACGACAAGAAGCTTTCGTCTGCCAAAGACAAACTGTA
MAVS-R	TGATGGATATCTGCAGAATTCCAGCCTCTGTCCTGTCTACTTCATG
MAVS-3'UTR-WT-F	CTAGCTAGCCGTATGGTGCCTTATTG
MAVS-3'UTR-WT-R	TGCTCTAGACAGCCTCTGTCCTGTCTACT
MAVS-3'UTR-mVenus-F	CCCAAGCTTGCTAGCGTATGGTGCCTTATTGG
MAVS-3'UTR-mVenus-R	CGCGGATCCCAGCCTCTGTCCTGTCTACT
MAVS-3'UTR-MT-F	CACCAAATCTACAgaCTGATTGTTAAGTTCTGGGTTGAGAT
MAVS-3'UTR-MT-R	CAGtcTGTAGATTTGGTGTCTGCTCGATCGGGT
MIR122HG-F	ACTATAGGGAGACCCAAGCTTTTAGCTGTTTGGAAAGACTCCGA
MIR122HG-R	TGATGGATATCTGCAGAATTCGCAGAAGTAAGTGGGTCACGCT
MIR122HG-MT-F	AGTACGTCTGCGCACTACGCTGTCCTCTGTATCAAACGCCATT
MIR122HG- MT-R	GTAGTGCGCAGACGTACTGGCAGCTCTGGAGGACAGCATTTAC
*Dre*MIR122HG-F	ACTATAGGGAGACCCAAGCTTAGGTTGCCCATCCGCCAG
*Dre*MIR122HG-R	TGATGGATATCTGCAGAATTCGCAGAAGTAAGAGGGTCACGCT
*Lcr*MIR122HG-F	ACTATAGGGAGACCCAAGCTTAGGTCGTCCATCCACCAGTTT
*Lcr*MIR122HG-R	TGATGGATATCTGCAGAATTCGCAGAAGTAAGTGGGTCACGCT
*Dre*MAVS-3'UTR-WT-F	CGCGAGCTCTGAGAATGTCTAACAGGCAC
*Dre*MAVS-3'UTR-WT-R	TGCTCTAGAAAGAACAGGAACAATCAAGA
*Dre*MAVS-3'UTR-MT-F	CTTGAcatTTCCTGTTCTTAAAGATTTTTACAAATG
*Dre*MAVS-3'UTR-MT-R	GAACAGGAAatgTCAAGATTGACCAGTACACAAAAAAG
*Lcr*MAVS-3'UTR-WT-F	CCGCTCGAGACCCTCCAGACCTTTGAT
*Lcr*MAVS-3'UTR-WT-R	GTCGACGTCGACGCTCCTCGTTAATCCTCA
*Lcr*MAVS-3'UTR-MT-F	CcatTTTTAAGTTCTGGGTTCAGATCTCAGGT
*Lcr*MAVS-3'UTR-MT-R	CCAGAACTTAAAAatgGAAGTGTAGATTTGGTGTGAGCTCG
